# Predictors of surgical management of high grade blunt splenic injuries in adult trauma patients: a 5-year retrospective cohort study from an academic level I trauma center

**DOI:** 10.1186/s13037-020-00257-3

**Published:** 2020-08-03

**Authors:** Thomas M. P. Nijdam, Roy Spijkerman, Lilian Hesselink, Luke P. H. Leenen, Falco Hietbrink

**Affiliations:** Department of Traumasurgery, University Medical Center Utrecht, Heidelberglaan 100, 3584CX Utrecht, The Netherlands

**Keywords:** Intra-abdominal, Trauma, Blunt, Spleen, Splenic

## Abstract

**Backgrounds:**

Splenic injury accounts for 40% of all injuries after blunt abdominal trauma. Blunt splenic injury in hemodynamically unstable patients is preferably treated by splenectomy. Nowadays hemodynamically stable patients with low grade splenic injuries are mostly treated by non-operative management (NOM). However no consensus exists about the management of high grade splenic injuries in hemodynamically stable patients. Therefore the aim of this study was to analyze patients with high grade splenic injuries in our institution.

**Methods:**

We retrospectively included all patients with a splenic injury presented to our level I trauma center during the 5-year period from January 1, 2012, until December 31, 2017. Baseline characteristics, data regarding complications and mortality were collected from the electronic patient registry. Patients were grouped based on splenic injury and the treatment they received.

**Results:**

A total of 123 patients were included, of which 93 (75.6%) were male with a median age of 31 (24–52) and a median injury severity score of 27 (17–34). High grade injuries (*n* = 28) consisted of 20 Grade IV injuries and 8 grade V injuries. Splenectomy was required in 15/28 (53.6%) patients, of whom all remained hemodynamically unstable after resuscitation, including all grade V injuries. A total of 13 patients with high grade injuries were treated with spleen preserving therapy. Seven of these patients received angio-embolization. One patient went for laparotomy and the spleen was treated with a hemostatic agent. Secondary hemorrhage was present in 3 of these patients (initial treatment: 1 embolization/ 2 observational), resulting in a success rate of 76.9%. There is no mortality seen in patient with high grade splenic injuries.

**Conclusion:**

Non-operative treatment in high grade splenic injuries is a safe treatment modality in hemodynamically stable patients. Hemodynamic status and peroperative bleeding, not injury severity or splenic injury grade were the drivers for surgical management by splenectomy. This selected cohort of patients must be closely monitored to prevent adverse outcomes from secondary delayed bleeding in case of non-operative management.

## Introduction

The spleen in the most frequently injured intra-abdominal organ in blunt abdominal trauma [1, 2]. Until the mid-sixties the only treatment used for blunt splenic injury (BSI) was a splenectomy [3, 4]. In the following years it became more common to preserve the spleen and nowadays the most common approach is non-operative management (NOM) [5–7]. Spleen preserving treatment is the preferred treatment option, to preserve the immunological and filter function of the patients spleen [8]. Spleen preservation prevents the patient from the overwhelming post-splenectomy infection (OPSI)-syndrome with reported mortality rates up to 70% [9, 10]. However, Spleen preservation is not always an option since it can lead to life-threatening hemorrhages [11]. The most important prerequisite for successful NOM is adequate patient selection [12–15]. Hence, it is of utmost importance to choose the right management for the right patient.

Nowadays, the presence of hemodynamic instability is considered the only absolute contraindication for NOM [3, 16]. In hemodynamically stable patients with low grade splenic injuries (≤ grade 3), the risk of secondary hemorrhage is so little that observational management is the standard [17]. However no consensus exists about the management of high grade splenic injuries (> grade 3) in hemodynamically stable patients. In some institutions, splenectomy is the preferred treatment option, whereas in other institutes all of these patients are treated by spleen preserving treatment options [12, 14, 18, 19]. Preferably, when a blush is seen on contrast enhanced computed tomography (CT)-scan, either central or partial angio-embolization(AE) is performed [20–22]. When no blush is seen and there is absence of other indications for laparotomy, the remaining spleen preserving options are observational management or surgery with hemostatic agents, Vicryl mesh or splenorrhaphy [23]. Although an increasing body of evidence supports spleen preservation for high grade injuries in the hemodynamically stable patient, there is limited literature regarding the safety of this approach [24, 25]. Therefore, the aim of this study was to analyze the treatment of high grade spleen injuries in our Dutch level I trauma center.

## Materials and methods

### Study design

A retrospective, observational study was performed to investigate the management of blunt splenic injuries in the University Medical Center Utrecht (UMCU). For this analysis, a waiver was provided by the institutional medical ethics committee. In addition, in line with the academic hospital policy, an opt-out procedure is in place for the use of patient data for research purposes. The process and storage of data are in accordance with privacy and ethics regulations.

### Patients

The UMCU is a regional referral center (and level I trauma center) of the region ‘Midden-Nederland’ that inhabits 2.2 million people. The UMCU is connected with several referral hospitals for the transfer of trauma patients. From the National Trauma Registration database we retrospectively identified patients presented to the UMCU. This national trauma registration contains data from patients who were treated in an emergency department (ED) within 48 h after the accident and subsequently admitted to a hospital for treatment or patients who died in the ED. Included were all patients ≥18 years in the UMCU or transferred from a referral hospital to the UMCU, diagnosed with splenic injury during the 5-year period from January 1, 2012 until December 31, 2017. Patients were excluded when aged < 18 years, deceased on arrival or in the ED and when splenic injury was iatrogenic.

All patient charts and follow-up files were reviewed and patient characteristics, trauma characteristics, diagnostic workup, treatment and outcome were documented. Patient and trauma characteristics included age in years, gender, transfer from another hospital, mechanism of injury, systolic blood pressure (SBP) in millimeter of mercury, pulse rate (PR) in beats per minute, respiration rate (RR) in number of breaths per minute, Glasgow Coma Score (GCS), serum hemoglobin (Hb) in millimole per liter, pH, lactate (mmol/l). Injuries were graded based on American Association for Surgery of Trauma organ injury grading scales. All injuries were scored according to the abbreviated injury scale (AIS) by an authorized registrar by analyzing the CT-scan or after abdominal exploration [26]. Currently used in the UMC Utrecht, is a Philips iCT 256-slices CT-scanner (Philips Medical Systems, Best, the Netherlands) for full body CT-angiogram [27]. Patients received split bolus high flow intravenous contrast administration during CT-scanning. The injury severity score (ISS) was calculated per patient after dismissal [28]. The ISS was calculated with version ‘98 until January 13, 2015, afterwards the ISS’08 was used. Success rate was defined as treatment without secondary hemorrhage or the need for re-intervention.

Patients were grouped based on splenic injury and the treatment they received. Groups were divided between patients who underwent splenectomy or either treated spleen preserving therapy; which consisted of observational treatment, splenic angio-embolization or laparotomy with spleen preserving procedures. The patients in group A suffered from high grade (AIS grade 4 and 5) splenic injuries and were treated with splenic preserving treatment. Group B consisted of patients with high grade splenic injury who underwent splenic extirpation. Group C consisted of patients with low grade splenic injury (AIS grade 1, 2 and 3) who were treated with spleen preserving procedures. Patients with low grade splenic injuries who received splenectomy were included in group D.

### Statistical analysis

All data were analyzed with SPSS version 25.0.0.2 (IBM Corporation, NY, United States). The distribution of continuous variables was assessed with the use of the Kolmogorov-Smirnov test and visual inspection of Q-Q plots. Results were presented as median with interquartile range (IQR), because all the data were not normally distributed. Comparison of baseline and outcome characteristics between groups was performed with a Chi-square/Fisher’s exact test or a Mann-Whitney U test, as indicated. Statistical significance was defined as a *p*-value < 0.05.

## Results

### Patient characteristics

**Fig. 1:**
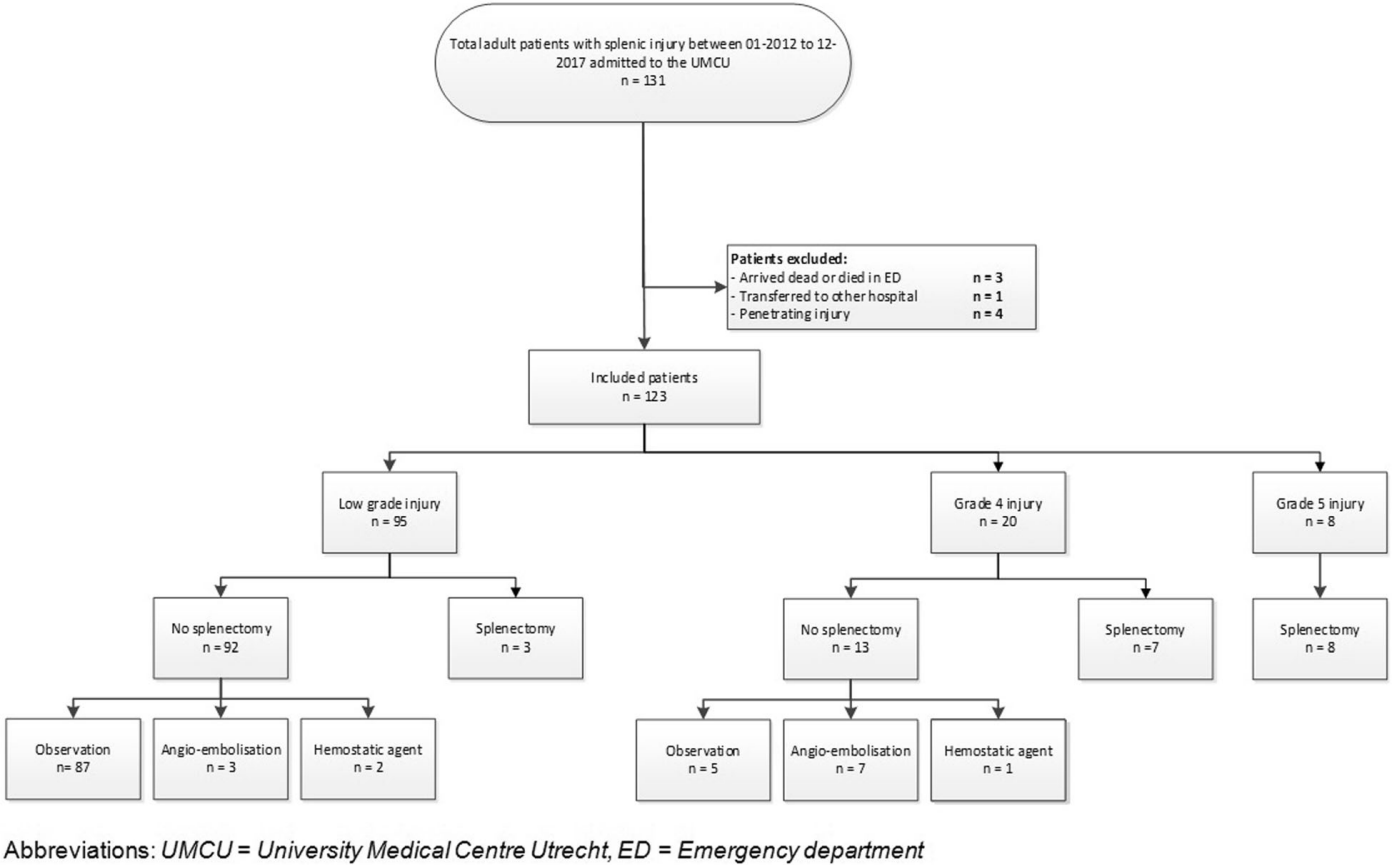
Flowchart splenic injury

A total of 181 patients with splenic injuries were identified from the trauma registry. After exclusion, 123 patients were included for final analysis (Fig. 1). The study population consisted of 93 males and 30 females with a median age of 31 (24–52) and a median ISS of 27 (17–34). The majority of the patients were injured by a traffic accident (*n* = 95) or fall from height (*n* = 19). Baseline characteristics of patients with high grade splenic injury are shown in Table 1. High grade injuries (*n* = 28) consisted of 20 Grade IV injuries and 8 grade V injuries. Of all patients with high grade splenic injuries (*n* = 28), splenectomy was performed in 15 patients (53%)(group B), 7 grade IV injuries and all 8 grade V injuries. All of these 28 patients (*n* = 15) remained hemodynamically unstable. As a result, 13 were treated with spleen preserving therapy (group A). Seven of these patients underwent AE. Of these patients, a splenic artery blush on CT-scan was seen in 3 patients. Five patients underwent AE with no splenic artery blush, but because of large amount of free abdominal fluid and a significant injury on CT-scan.

**Table 1:** Baseline characteristics

	Total high grade (*N* = 28)	High grade splenic injuries		
		Group A: Initially no splenectomy (*N* = 13)	Group B: Initial splenectomy (*N* = 15)	***P***-value
Age at trauma, years	39 (25–52)	40 (29–51)	37 (23–55)	0.580
Gender (M/F)	25/3	11/2	14/1	0.457
Injury Severity Score	34 (27–44)	29 (23–35)	34 (29–57)	0.096
**AIS spleen**				
Grade 4	20	13	7	na
Grade 5	8	0	8	
Hemodynamically stable (N/Y)	18/10	3/10	15/0	**< 0.001**
Blush on CT	7	3	4	0.827
**Treatment**				
Conservative	5	5	0	na
Embolization	7	7	0	
Hemostatic agent + lap.	1	1	0	
Splenectomy	15	0	15	
**Mechanism of injury**				
Motor cyclist	11	5	6	na
Pedal cyclist	4	2	2	
Pedestrian	0	0	0	
Car occupant	8	6	2	
Fall	3	0	3	
Other	2	0	2	
Transfer from other hospital	5	3	2	0.502
Glasgow Coma Scale	15 (9–15)	15 (14–15)	15 (3–15)	**0.019**
**Total blood transfusion**				
Erythrocyte concentrate	4 (0–6)	1 (0–4)	5 (2–8)	**0.005**
Fresh frozen plasma	3 (0–5)	0 (0–4)	4 (2–9)	**0.014**
Thrombocytes	0 (0–1)	0 (0–1)	1 (0–2)	**0.013**
Pulse rate	89 (78–114)	81 (69–110)	90 (82–129)	0.240
Systolic blood pressure	107 (95–127)	112 (95–132)	103 (95–121)	**0.279**
Respiratory rate	20 (17–23)	20 (17–22)	19 (17–25)	0.699
Serum Hemoglobin	7.7 (7.0–8.7)	8.7 (7.5–9.1)	7.2 (6.5–8.2)	**0.019**
Platelets	206 (168–271)	204 (169–284)	208 (160–254)	0.696
Lactate	2.9 (2.1–4.7)	2.1 (1.7–3.6)	4.1 (2.7–5.4)	**0.016**
Leukocytes	15.8 (11.4–20.9)	14.1 (9.8–21.2)	17.3 (12.7–20.9)	0.558

All variables are in total amount, median (IQR) or median (range). Thrombocytes contain 5 units/ transfusion

*Abbreviations*: *AIS* Abbreviated injury scale, *M/F* Male/Female, *N/Y* No/Yes, *lap.* laparotomy

Low grade injuries were presented in Supplement 1, 95 patients of whom 88 patients had a grade II and 42 patients had a grade III injury. Of all patients with low grade injuries (*n* = 95), spleen preserving management was performed in 92 patients (97%) (group C). A total of 90 could be treated by NOM. There were 7 patients (8%) with splenic artery blushes on CT-scan, of whom 3 patients (3%) were treated with AE. In two patients peroperative hemostatic agent was used to treat the bleeding. Three out of 95 patients (8%) with low grade injuries required splenectomy (group D) due to severe concomitant abdominal injury resulting in massive blood loss and hemodynamic instability. In these patients the surgeon choose for damage control and removed the spleen instead of packing.

Differences in baseline characteristics are described in Table 1. Patient with high grade injuries treated with a splenectomy had a significant lower GCS (15 (4–15) vs. 15 (3–15), *p* = 0.019) and a higher lactate (2.1 (1.7–3.6) vs. 4.1(2.7–5.4), *p* = 0.016) than patients with high grade injuries treated by spleen preserving treatment. All of the patients with a high grade splenic injury in the splenectomy group were hemodynamically unstable, this was visible by significant more transfusion requirements of ECs (*p* = 0.005), FFP’s (*p* < 0.014) and thrombocytes (*p* < 0.013).

An overview of all concomitant abdominal injuries is shown in Table 2. The median AIS of severe other abdominal injuries was not significantly different between patients with high grade splenic injuries who underwent a splenectomy and those who did not (3 (3–4)* vs 3 (3–4)*, *p* = 0.456). A total of 24 of the 92 (26%) patients treated with spleen preserving treatment had other severe abdominal injuries. This was significantly higher than in patients with high grade splenic injuries, in which 6/28 patient had other severe abdominal injuries (21%). The most common severe other abdominal injury was high grade renal injury and high grade hepatic injury.

**Table 2:** Concomitant abdominal injuries

	Total high grade (*N* = 28)	High grade splenic injury		
		Group A: Initially no splenectomy (*N* = 13)	Group B: Initial splenectomy (*N* = 15)	***P***-value
**Patients with other severe abd. injuries:**	6 (21.4)	3 (23.1)	3 (20.0)	0.690
AIS severe other abd. injuries	3 (3–4)	3 (3–4)^a^	3 (3–4)^a^	0.456
AIS all other abd. injuries	0 (0–2)	0 (0–2)	0 (0–2)	0.452
**Severe abdominal injuries:**				
Hepatic injury	3	2	1	na
Renal injury	2	1	1	
Urinary tract	0	0	0	
Vascular	1	0	1	
Diaphragm	0	0	0	
Hollow viscus	3	1	2	
**Interventions indication for severe trauma other than spleen:**				
Embolization	0	0	0	na
Laparotomy	3	1	2	
Laparotomy + packing	1	0	1	
Laparotomy + fibrin sealant	0	0	0	
Laparotomy + splenic mesh	1	1	0	

All variables are in total amount (%), median (IQR) or median (range)^a^

*Abbreviations*: *AIS* Abbreviated injury scale, *abd* abdominal, *na* not applicable

### Clinical outcomes

Outcome measurements from the high grade injuries are shown in Table 3. Complications were observed in 7 of the 123 patients (5.7%). Six of these patients had high grade splenic injuries (group A and group B, 6/25, 24%).

**Table 3:** Outcome measurements

	Total high grade (*N* = 28)	High grade splenic injury		
		Group A: Initially no splenectomy (*N* = 13)	Group B: Initial splenectomy (*N* = 15)	***P***-value
**Patients with spleen related complications:**	6	3	3	0.843
**Spleen related complications:**				
No complications	22	10	12	na
Secondary hemorrhage	3	3	0	
Wound infection	2	0	2	
Abscess	1	0	1	
**Surgical (re-)interventions:**				
Hemostatic agent	0	0	0	na
Mesh	1	1	0	
Splenectomy	2	2	0	
Length of hospital stay in days	15 (8–24)	14 (8–21)	15 (8–29)	0.945
Length of ICU stay in days	6 (1–16)	6 (1–14)	6 (1–22)	0.904
Ventilation days	8 (3–15)	8 (3–22)	11 (3–15)	0.748
Mortality	1	0	1	na

All variables are in total amount, median (IQR) or median (range)

*Abbreviations*: ^*a*^ AIS grade spleen, *ICU* intensive care unit, *na* not applicable

Two high grade splenectomy patients developed wound infections, they recovered with antibiotics. Three patients with high grade injuries developed a secondary hemorrhage after conservative treatment. Two patients with secondary hemorrhage were splenectomized, the other patient received a Vicryl mesh around the spleen for compression and stability.

Two patients developed abscesses, one of them with low grade splenic injury and one with high grade splenic injury. Both were treated with splenectomy. The two patients received a CT or ultrasound guided drainage of the abscess. Of all patients initially treated with spleen preserving treatment, spleen preservation was successful in 103 of 105 (98%) patients. Patients with low grade injuries treated by NOM did not develop any spleen related complications.

A total of 7 patients died (5.7%). None of the patients died because of their splenic injury. Three patients died due to severe brain injuries, 3 patients died of hypovolemic shock and 1 patient died because of a sepsis due to a large gastric perforation what resulted in gastric necrosis.

### Case description of patients with a secondary hemorrhage

In group A (high grade injuries managed with spleen preserving therapy), there were three patients with secondary hemorrhage. One patient with a grade IV splenic injury, was hemodynamically stable and treated conservatively at first, but later became hemodynamically unstable with heavy abdominal pain and tensions of 70/40 mmHg. A secondary hemorrhage was suspected and a splenectomy was performed.

The second patient developed a secondary hemorrhage after AE was performed for a grade IV injury. Four hours after coiling, the patient remained extremely painful in the abdomen combined with low tensions, low pulse and with a prolonged prothrombin time of 16.9 before resuscitation (2 packed cells and 2 Fresh Frozen Plasma). After the Hb dropped to 5.6 (from 6.8), the patient went for laparotomy for exploration of the abdominal cavity and a Vicryl mesh was applied around the spleen for compression.

The third patient in group A with a splenic injury grade IV had a secondary hemorrhage 9 days after trauma. There were no signs of hemorrhage directly after trauma on CT-scan, however patient underwent rib fixation (left rib 4 to rib 10) on the 8th day after trauma. One day after rib fixation, hemoglobin levels dropped to 3.0 (from 7.1) and a tension of 90/60 mmHg was measured. The patient went for emergent laparotomy and the spleen was removed due to active splenic bleeding.

### Case description of hemodynamic unstable patients with splenic preservation

Three patients in group A who were treated conservatively for their splenic injury, did require operative management (laparotomy) for concomitant abdominal injuries in combination with persistent hemodynamic instability. Two patients only needed abdominal packing and in one patient a mesh was applied around the spleen.

One hemodynamically unstable patient with free fluid on FAST echo after blunt trauma underwent a laparotomy. A large hematoma of the mesentery was seen, a large amount of free blood was evacuated and a large laceration of the serosa from the transverse colon was sutured. After this, the patient stabilized and as the spleen (grade IV injury) was partly ruptured, a Vicryl mesh was placed around the spleen. There was no suspicion for active hemorrhage of the spleen during or after surgery.

The second persistently hemodynamically unstable patient with free fluid on FAST exam after blunt trauma received emergency laparotomy. Initially, after the peritoneum was opened a large amount of blood was evacuated and a large laceration from the liver was seen with necrotic tissue on the right side of the liver. The liver was packed multiple times and because of oozing hemorrhage a hemostatic agent was placed on the liver tissue, which controlled the bleeding. The splenic injury was classified as grade IV, no active hemorrhage after depacking was seen and splenic salvation could be achieved.

The third patient with splenic preservation and hemodynamic instability during admittance after blunt trauma showed free abdominal fluid on FAST echo and a laparotomy was performed. Initially, the abdomen was packed in all quadrants. The splenic injury was estimated grade IV, without active blood loss and therefore without the need of immediate intervention besides packing. A second look laparotomy on day 2 showed no active abdominal hemorrhage and the spleen remained preserved.

## Discussion

This study retrospectively analyzed all patients with splenic injuries admitted to our institution for the last 5 years. We found that a total of 105/123 (85.4%) patients could be treated by spleen preserving treatment, of these 105 patients were 93 (88.6%) patients low grade and 13 (12.4) with high grade splenic injuries. In 15 patients with high grade injuries splenectomy was needed. In all 15/15 patients the reason for splenectomy was hemodynamic instability. The splenic salvage rate of spleen preserving treatment in patients with low grade injuries was 100 and 85% in patient with a high grade injury.

In line with our study, it has been frequently reported in the literature that spleen preserving treatment is safe for low-grade injures [25, 29, 30]. However, since the literature on spleen preserving treatment for high-grade injuries is limited, we analyzed these injuries in more detail in addition to the low grade injuries. In total we found 3 secondary hemorrhages in patients with high grade splenic injuries treated by NOM. However, all hemorrhages were quickly diagnosed and treated, so that it did not cause any additional morbidity or mortality. Hemodynamically stable patients were always treated with spleen preserving treatment.

A possible reason for the high success rate of spleen preserving treatment in patients with high grade injuries, is the addition of AE as treatment. This treatment was described for the first time in 1995 [31]. In this study, 60 patients underwent AE. In a Norwegian study, Gaarder et al. described a success rate of 96% in the NOM group after introduction of AE [22]. With a success rate of 97.1% in 102 out of 105 patients, the success rate found in our study was similar to these results. The statistically significant improvement in splenic salvage in Haan et al. showed the relevance of the addition of AE in non-operative treatment [17]. The study compares their data with the EAST study from Peitzman et al., in which NOM only consisted of observational management [32]. Peitzman described a 10.8% failure rate of non-operative management, in which they included observational management without the use of AE. With a failure rate of NOM < 3% in our study, the addition of AE to the treatment of splenic injury, as concluded by Haan et al., is a possible explanation of the lower failure rate compared to Peitzman’s study [17, 32].

Another possible reason for high splenic salvation is adequate fluid resuscitation of trauma patients for hemostasis. Without appropriate fluid resuscitation, hemorrhage, including splenic hemorrhage, will not be controlled. Inadequate resuscitation may even disrupt the clot and lead to more bleeding in combination with tissue hypoxia due to inadequate global oxygen delivery [33]. In the UMCU the resuscitation protocol with a 5:5:1 ratio (Packed cells: Fresh frozen plasma: Thrombocytes (5 units in 1 pack)) is used for trauma patients, corresponding to a ratio of 1:1:1 [34–36]. Splenic preservation was also achieved in a few hemodynamically unstable patients with high grade injuries. As described in the case descriptions in the results section, when hemodynamic stability was achieved by managing other bleeding sources and if there was no active splenic hemorrhage (the next day) after abdominal packing, the spleen remained in place. This was only possible when the patients stabilized during surgery and the other abdominal injuries were clearly the cause of the instability and could be adequately managed.

The main limitation of our study is that this was a retrospective study. Due to this study design we were not able to rate all injuries with the same ISS. Within the time period of our study the UMCU changed the ISS from the 98 version to the more up to date ISS 08. Data were not available to recalculate the ISS and the slightly different ISS were therefore taken the same and not recalculated. Another disadvantage of a retrospective design is that there were some missing data in the national trauma registry. Data that were missing could be easily traced, if properly documented, in the UMCU’s electronic health record (EHR). Another limitation is the low number of patients with high grade injuries in this study. Missing data from this small group could be gained directly from the UMCU’s EHR. Therefore, a strength of this study is that there were only very few missing data because of the addition of information from the EHR to the data from the National Trauma Registration.

**Fig. 2:**
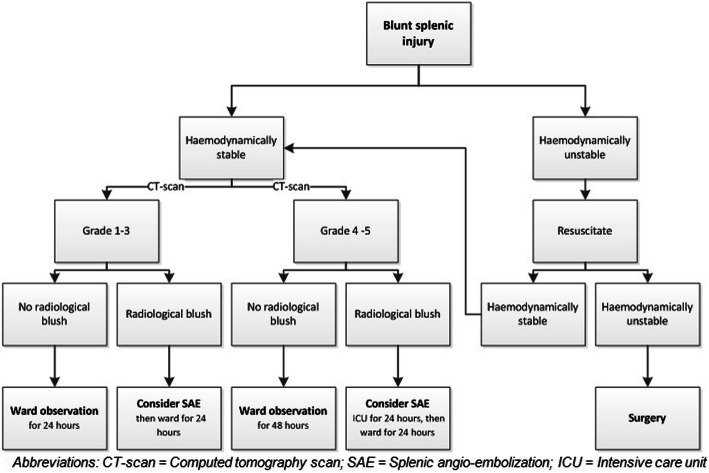
Suggested splenic injury protocol

To help support clinical decision making, we created a protocol (Fig. 2) for patients with blunt splenic injuries. We divided the groups in hemodynamic stable and unstable patients. The hemodynamically unstable patient should be treated with a splenectomy when resuscitation fails. The hemodynamic stable patient can be safely treated with NOM. However, for both high grade and low grade splenic injuries, AE should be considered if a contrast blush is seen on CT-scan. Otherwise, conservative management can be chosen. In this case, it is important to monitor the patient closely for at least 24 h in case of low grade injuries or 48 h in case of high grade injuries. Moreover, for the best clinical outcome of NOM, adequate resuscitation is of utmost importance.

## Conclusion

Non-operative treatment in high grade splenic injuries is a safe treatment modality in hemodynamically stable patients. Hemodynamic status and peroperative bleeding, not injury severity or splenic injury grade were the drivers for surgical management by splenectomy. This selected cohort of patients must be closely monitored to prevent adverse outcomes from secondary delayed bleeding in case of non-operative management.

## Supplementary information

**Additional file 1 Supplement 1.** Baseline Low grade. **Supplement 2.** Concomitant abdominal injuries low grade. **Supplement 3.** Outcome measurements low grade.

## Data Availability

The datasets during and/or analysed during the current study available from the corresponding author on reasonable request.
