# Supplementary material for: Predictors of surgical management of high grade blunt splenic injuries in adult trauma patients: a 5-year retrospective cohort study from an academic level I trauma center
Source: Patient Saf Surg. 2020 Aug 3;14:32. doi: 10.1186/s13037-020-00257-3 (PMC7398213; doi:10.1186/s13037-020-00257-3)
Supplement: Supplementary file 1 — Additional file 1 Supplement 1. Baseline Low grade. Supplement 2. Concomitant abdominal injuries low grade. Supplement 3. Outcome measurements low grade. [file 13037_2020_257_MOESM1_ESM.docx]

**Supplement 1. Baseline Low grade**

|  | **Total**  **Low & High grade**  (N=123) | **Low grade splenic injuries** | | |
| --- | --- | --- | --- | --- |
|  |  | **Group C:**  Initial no splenectomy  (N=92) | **Group D:**  Initial splenectomy  (N=3) | **P-value** |
| Age at trauma, years | 31 (24-52) | 30 (23-52) | 46 (30-83)* | 0.194 |
| Gender (M/F) | 93/30 | 66/26 | 2/1 | 1.000 |
| Injury Severity Score | 27 (17-34) | 22 (14-34) | 26 (22-50)* | 0.311 |
| **AIS spleen**  Grade 2  Grade 3  Grade 4  Grade 5 | 73  22  20  8 | 72  20  0  0 | 1  2  0  0 |  |
| Hemodynamically stable (N/Y) | 35/88 | 14/76 | 3/0 | **0.006** |
| Blush on CT | 14 | 7 | **0** | na |
| **Mechanism of injury**  Motor cyclist  Pedal cyclist  Pedestrian  Car occupant  Fall  Other | 29  16  7  43  19  9 | 18  12  6  33  16  1 | 0  0  1  2  0  0 | na |
| Transfer from other hospital | 10 | 5 | 0 | na |
| Glasgow Coma Scale | 15 (8-15) | 15 (4-15) | 12 (3-14)* | 0.222 |
| **Total blood transfusion**  Erythrocyte concentrate  Fresh frozen plasma  Thrombocytes | 0 (0-4)  0 (0-4)  0 (0-0) | 0 (0-1)  0 (0-0)  0 (0-0) | 10 (8-13)*  12 (0-13)*  2 (0-2)* | **0.001**  **0.028**  **0.023** |
| Pulse rate | 85 (76-110) | 85 (76-105) | 100 (80-105)* | 0.504 |
| Systolic blood pressure | 121 (105-135) | 126 (110-137) | 110 (100-113)* | 0.118 |
| Respiratory rate | 18 (15-22) | 18 (15-22) | 10 (8-20)* | 0.100 |
| Serum Hemoglobin | 8.2 (7.4-9.0) | 8.5 (7.7-9.1) | 6.5 (6.3-8.4)* | **0.088** |
| Platelets | 241 (192-288) | 248 (197-300) | 168 (131-249)* | 0.115 |
| Lactate | 2.3 (1.7-3.6) | 2.2 (1.6-3.5) | 1.9 (1.5-3.6)* | 0.756 |
| Leukocytes | 14.7 (10.2-20.38) | 13.8 (9.6-20.1) | 15.5 (14.5-22.0)* | 0.437 |

*All variables are in total amount, median (IQR) or median (range)*. Thrombocytes contain 5 units/ transfusion. Abbreviations: AIS= Abbreviated injury scale, (M/F) = (Male/Female), (N/Y) = (No/Yes), MVA =Motor vehicle accident, na = not applicable*

**Supplement 2. Concomitant abdominal injuries low grade**

|  | **Low & High grade** | **Low grade splenic injury** | | |
| --- | --- | --- | --- | --- |
|  | **Total**  (N=123) | **Group C:**  Initially no splenectomy  (N=92) | **Group D:**  Initial splenectomy (N=3) | **P-value** |
| **Patients with other severe abd. injuries:** | 30 (24.4) | 23 (25.0) | 1 (33.3) | 1.000 |
| AIS severe other abd. injuries  AIS all other abd. injuries | 4 (3-4)  0 (0-2) | 3 (3-4)  0 (0-2) | 4  0 (0-2) | 0.473  0.962 |
| **Severe abdominal injuries:**  Hepatic injury  Renal injury  Urinary tract  Vascular  Diaphragm  Hollow viscus | 15  14  1  4  2  7 | 12  12  1  2  1  4 | 0  0  0  1  1  0 | na |
| **Interventions indication for severe trauma other than spleen:**  Embolization  Laparotomy  Laparotomy + packing  Laparotomy + fibrin sealant  Laparotomy + splenic mesh | 2  11  5  2  1 | 2  9  3  2  0 | 0  0  1  0  0 | na |

*All variables are in total amount (%), median (IQR) or median (range)*. Abbreviations: AIS= Abbreviated injury scale, abd = abdominal, na = not applicable*

**Supplement 3. Outcome measurements low grade**

|  | **Low & High grade** | **Low grade splenic injury** | | |
| --- | --- | --- | --- | --- |
|  | **Total**  (N=123) | **Group C:**  Initially no splenectomy  (N=92) | **Group D:**  Initial splenectomy (N=3) | **P-value** |
| **Patients with spleen related complications:** | 7 | 0 | 1 | na |
| **Spleen related complications:**  No complications  Secondary hemorrhage  Wound infection  Abscess | 116  3  2  2 | 92  0  0  0 | 2  0  0  1 | na |
| **Surgical (re-)interventions:**  Hemostatic agent  Mesh  Splenectomy | 0  1  2 | 0  0  0 | 0  0  0 | na |
| Length of hospital stay in days | 12 (4-21) | 11 (4-20) | 29 (4-30)* | 0.287 |
| Length of ICU stay in days | 5 (2-11) | 5 (2-10) | 4 (1-11)* | 0.780 |
| Ventilation days | 5 (2-11) | 6 (2-10) | 4 (0-8)* | 0.407 |
| Mortality | 7 | 5 | 1 | 0.180 |

*All variables are in total amount, median (IQR) or median (range)*. Abbreviations: * =AIS grade spleen, ICU = intensive care unit, na = not applicable*
